# MicroRNA-21 links epithelial-to-mesenchymal transition and inflammatory signals to confer resistance to neoadjuvant trastuzumab and chemotherapy in HER2-positive breast cancer patients

**DOI:** 10.18632/oncotarget.5495

**Published:** 2015-10-07

**Authors:** Leticia De Mattos-Arruda, Giulia Bottai, Paolo G. Nuciforo, Luca Di Tommaso, Elisa Giovannetti, Vicente Peg, Agnese Losurdo, José Pérez-Garcia, Giovanna Masci, Fabio Corsi, Javier Cortés, Joan Seoane, George A. Calin, Libero Santarpia

**Affiliations:** ^1^ Vall d'Hebron Institute of Oncology, Vall d'Hebron University Hospital, Universitat Autònoma de Barcelona, Barcelona, Spain; ^2^ Oncology Experimental Therapeutics Unit, IRCCS Humanitas Clinical and Research Institute, Rozzano, Milan, Italy; ^3^ Molecular Oncology Group, Vall d'Hebron Institute of Oncology, Barcelona, Spain; ^4^ Division of Pathology, IRCCS Humanitas Clinical and Research Institute, Rozzano, Milan, Italy, University of Milan, Milan, Italy; ^5^ Department of Medical Oncology, VU University Medical Center, Amsterdam, The Netherlands; ^6^ Cancer Pharmacology Laboratory, AIRC Start-Up Unit, University of Pisa, Pisa, Italy; ^7^ Pathology Department, Vall d'Hebron University Hospital, Universitat Autonoma de Barcelona, Barcelona, Spain; ^8^ Division of Oncology and Hematology, IRCCS Humanitas Clinical and Research Institute, Rozzano, Milan, Italy; ^9^ Deparment of Clinical and Biomedical Sciences “Luigi Sacco”, University of Milan, Milan, Italy; ^10^ Ramon y Cajal University Hospital, Madrid, Spain; ^11^ Institució Catalana de Recerca i Estudis Avançats (ICREA), Barcelona, Spain; ^12^ Department of Experimental Therapeutics, The University of Texas MD Anderson Cancer Center, Houston, Texas, USA; ^13^ Center for RNA Interference and Non-Coding RNAs, The University of Texas MD Anderson Cancer Center, Houston, Texas, USA

**Keywords:** HER2-overexpressing breast cancers, microRNA-21, resistance to neoadjuvant trastuzumab-chemotherapy, epithelial-to-mesenchymal transition, tumor-associated immune response

## Abstract

Patients with primary HER2-positive breast cancer benefit from HER2-targeted therapies. Nevertheless, a significant proportion of these patients die of disease progression due to mechanisms of drug resistance. MicroRNAs (miRNAs) are emerging as critical core regulators of drug resistance that act by modulating the epithelial- to-mesenchymal transition (EMT) and cancer-related immune responses. In this study, we investigated the association between the expression of a specific subset of 14 miRNAs involved in EMT processes and immune functions and the response to neoadjuvant trastuzumab and chemotherapy in 52 patients with HER2-overexpressing breast tumors. The expression of only a single miRNA, *miR-21*, was significantly associated with residual disease (*p* = 0.030) and increased after trastuzumab-chemotherapy (*p* = 0.012). A target prediction analysis coupled with *in vitro* and *in vivo* validations revealed that *miR-21* levels inversely correlated with the expression of PTEN (rs = −0.502; *p* = 0.005) and PDCD4 (rs = −0.426; *p* = 0.019), which differentially influenced the drug sensitivity of HER2-positive breast cancer cells. However, PTEN expression was only marginally associated with residual disease. We further demonstrated that *miR-21* was able to affect the response to both trastuzumab and chemotherapy, triggering an IL-6/STAT3/NF-κB-mediated signaling loop and activating the PI3K pathway. Our findings support the ability of *miR-21* signaling to sustain EMT and shape the tumor immune microenvironment in HER2-positive breast cancer. Collectively, these data provide a rationale for using *miR-21* expression as a biomarker to select trastuzumab-chemotherapy-resistant HER2-positive breast cancer patients who may benefit from treatments containing PI3K inhibitors or immunomodulatory drugs.

## INTRODUCTION

HER2-overexpressing breast cancer represents approximately 20% of human breast cancers and is associated with an increased risk of tumor recurrence and reduced overall survival [1, 2]. The overexpression of HER2 enables the constitutive activation of growth factor signaling pathways and thereby serves as an oncogenic driver of breast cancer [3]. Despite the clinical benefit of HER2-targeted therapies, a substantial percentage of patients die of disease progression due to drug resistance [1–5]. Elucidating the molecular pathways involved in trastuzumab resistance has been difficult due to the variety of mechanisms of action of this drug. Multiple mechanisms of resistance have been proposed, including the deregulation of the phosphatidylinositol 3-kinase (PI3K)/AKT pathway, which is caused by activating mutations of *PIK3CA* or a loss of phosphatase and tensin homolog (PTEN), the reduced receptor-antibody binding affinity, and the increased signaling via alternative HER and non-HER family receptor tyrosine kinases [3–9]. However, final validations based on analyses of human tumor samples have been limited and are not entirely reproducible [3, 10]. Furthermore, trastuzumab in either the early or metastatic setting is administered with cytotoxic chemotherapy, which may be a further potential confounding factor in the search for specific predictive clinical biomarkers of drug resistance. Recently, additional pathways have been demonstrated to contribute to the resistance of HER2-positive breast tumors to trastuzumab and chemotherapy. Epithelial-to-mesenchymal transition (EMT) is a central biological event that allows cancer cells to avoid apoptosis and cellular senescence, which contributes to tumor progression [11]. Anti-HER2 and chemotherapeutic agents have been shown to increase the number of cells with mesenchymal traits and contribute to multidrug resistance in breast cancer [12–16]. Furthermore, the overexpression of *HER2* itself regulates EMT by directly activating downstream signaling, such as the PI3K pathway, and the induction of IL-6 release from cancer cells [15, 17]. Indeed, IL-6 has been demonstrated to activate STAT3/NF-κB signaling, which consequently sustains EMT in breast cancer, and to modulate the tumor microenvironment, linking inflammation to cancer progression and drug resistance [13, 17–21]. Therefore, a comprehensive molecular understanding of the pathways associated with resistance to trastuzumab and chemotherapy might greatly aid the development of more effective targeted therapies, whereas the discovery of clinical molecular predictors of response will allow a more personalized treatment approach for patients with HER2-amplified breast cancer.

In recent years, microRNAs (miRNAs), a class of small non-coding RNAs that regulate gene expression, have emerged as crucial regulators of the drug response that act by shaping the tumor immune microenvironment and modulating EMT [22–26]. Hence, identifying and targeting miRNAs that regulate pathways involved in tumor-associated inflammation and EMT may result in an effective integrative approach to overcome drug resistance in HER2-overexpressing breast cancers. In this study, we investigated the association and biological role of a specific subset of miRNAs involved in EMT and tumor-associated immune pathways. Specifically, we evaluated the response to neoadjuvant trastuzumab and chemotherapy in two cohorts of HER2-positive breast cancer patients. Furthermore, we identified the molecular mechanisms underlying miRNA-mediated drug resistance using *in vitro* and *in vivo* assays.

## RESULTS

### Overexpression of *miR-21* is associated with resistance to neoadjuvant trastuzumab-chemotherapy in HER2-positive breast cancer patients

**Figure 1:**
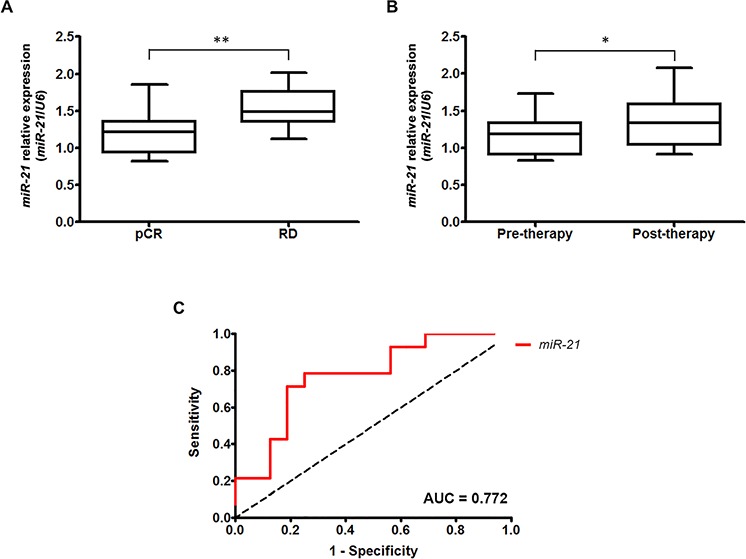
Overexpression of *miR-21* in HER2-overexpressing breast cancer is associated with trastuzumab and chemotherapy resistance **A.** *MiR-21* is significantly overexpressed in patients with RD compared with patients who achieved pCR (*p* = 0.012). **B.** *miR-21* expression was significantly higher in HER2-positive patients after trastuzumab and chemotherapy treatment (*p* = 0.016). *p < 0.05; **p ≤ 0.01. **C.** The ROC analysis demonstrated that *miR-21* expression was able to discriminate patients with pCR from those with RD with high accuracy (AUC = 0.772).

Based on a comprehensive literature review, we selected 14 functionally relevant miRNAs involved in the regulation of EMT and anti-tumor immune response and evaluated their clinical significance in primary HER2-positive (*n* = 22) and HER2-negative (*n* = 21) breast cancer patients who received neoadjuvant trastuzumab-chemotherapy or chemotherapy alone, respectively (Table 1, Supplementary Table 1). Within this subset of miRNAs, *miR-373* and *miR-18b* were not detectable in the majority of samples and were consequently excluded from subsequent analyses. We found that only a single miRNA, *miR-21*, was significantly differentially expressed between HER2-positive patients who achieved a pathological complete response (pCR) and patients with residual disease (RD) (*p* = 0.030) (Table 1). None of the miRNAs analyzed correlated with drug response in HER2-negative patients, suggesting that *miR-21* may be a specific biomarker of resistance for HER2-positive breast cancer (Table 1). A further analysis of an additional cohort of 30 HER2-positive breast cancers (Supplementary Table 1) confirmed that *miR-21* overexpression was associated with RD (*p* = 0.012) (Figure 1A), indicating that *miR-21* is indeed involved in resistance to neoadjuvant treatment consisting of trastuzumab and chemotherapy. Notably, although the baseline levels of *miR-21* predicted resistance to trastuzumab-chemotherapy treatment, we found that its expression was further upregulated after such therapy (*p* = 0.016) (Figure 1B). These data indicate that an additional increase in *miR-21* induced by the current therapy may sustain a molecular loop responsible for drug resistance in HER2-positive breast cancer. To further assess the ability of *miR-21* to discriminate the patients' responses to trastuzumab-based therapy, we performed a receiver-operating characteristic (ROC) analysis of the validation dataset. The results of this analysis confirmed that *miR-21* expression was able to discriminate patients who achieved a pCR from those with RD after neoadjuvant therapy with high accuracy (AUC = 0.772) (Figure 1C).

**Table 1:** MicroRNAs differentially expressed between responders and non-responders in HER2-positive and HER2-negative breast cancer patients

	HER2-positive		HER2-negative	
	Fold Change	*p*-value	Fold Change	*p*-value
*let-7b*	–1.177	0.621	–1.258	0.211
*miR-20a*	–1.116	0.812	–1.095	0.943
*miR-21*	–4.989	**0.030**	2.589	0.276
*miR-34a*	–1.427	0.373	1.275	0.562
*miR-106b*	1.825	0.337	–2.470	0.303
*miR-107*	–1.160	0.503	–1.020	0.905
*miR-145*	1.473	0.597	–1.270	0.930
*miR-155*	–1.968	0.198	1.975	0.190
*miR-181b*	–1.438	0.277	1.054	0.432
*miR-200b*	1.516	0.621	1.629	0.203
*miR-221*	–1.021	0.668	1.273	0.370
*miR-495*	1.197	0.817	–1.101	0.967

Significant *p*-values are given in bold

### *MiR-21* downregulates PTEN and PDCD4 contributing to trastuzumab-chemotherapy resistance in patients with HER2-overexpressing breast cancer

**Figure 2:**
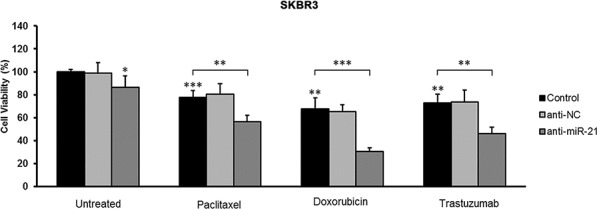
Effect of *miR-21* inhibition on the sensitivity of SKBR3 breast cancer cells to trastuzumab and chemotherapeutic agents Following transfection with a negative control or miR-21 inhibitor, SKBR3 cells were treated with paclitaxel (5 nM), doxorubicin (1 μM), or trastuzumab (10 μg/mL), and cell viability was assessed with an MTT assay. *MiR-21* inhibition alone and drug treatments reduced cell viability compared with untreated cells. Importantly, the inhibition of *miR-21* significantly increased the ability of both chemotherapeutic drugs and trastuzumab to reduce the viability of SKBR3 cells. All experiments were performed in triplicate. Error bars represent the S.D. *p < 0.05; **p ≤ 0.01; ***p ≤ 0.001.

To evaluate the role of *miR-21* in determining the drug response of HER2-positive breast cancers, we performed *in vitro* assays using a HER2-positive breast cancer cell line (SKBR3) treated with a *miR-21* inhibitor alone or in combination with chemotherapeutic drugs or trastuzumab. The *miR-21* inhibitor alone was able to decrease the viability of untreated cells (*p* = 0.042), but it was less effective than treatment with paclitaxel (*p* = 0.001), doxorubicin (*p* = 0.002), and trastuzumab (*p* = 0.002) (Figure 2). Notably, the inhibition of *miR-21* significantly increased the ability of paclitaxel (*p* = 0.005), doxorubicin (*p* = 0.001), and trastuzumab (*p* = 0.004) to reduce the viability of SKBR3 cells, indicating that *miR-21* is involved in both trastuzumab and chemotherapy resistance (Figure 2).

**Figure 3:**
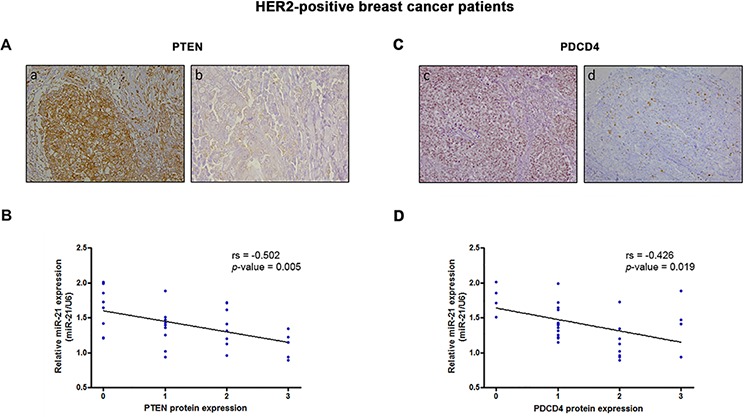
PTEN and PDCD4 protein levels correlate with *miR-21* expression in HER2-positive breast cancer The expression levels of PTEN (A, B) and PDCD4 (C, D) were assessed in the validation cohort by immunohistochemistry and graded as follows: low expression, score 0–1; high expression, score 2–3. **A.** Representative images of PTEN expression in samples with low (a) and high (b) *miR-21* expression. **B.** Correlation between *miR-21* expression and PTEN protein levels in HER2-positive breast cancers tissues (rs = −0.502; *p* = 0.005). **C.** Representative images of PDCD4 expression in samples with low (c) and high (d) *miR-21* expression. **D.** Correlation between *miR-21* expression and PDCD4 protein levels in HER2-positive breast cancers tissues (rs = −0.426; *p* = 0.019).

To investigate the molecular mechanisms underlying *miR-21*-mediated therapy resistance, we searched for predicted and validated target genes by combining several target prediction databases, including miRBase (http://microrna.sanger.ac.uk), TargetScan (http://www.targetscan.org), and Tarbase (http://diana.cslab.ece.ntua.gr/tarbase). Based on these analyses and the biological roles played in EMT and immune signaling [13, 27], PTEN and PDCD4 were selected for further evaluation. We found that inhibition of *miR-21* substantially increased PTEN and PDCD4 expression in SKBR3 cells (Supplementary Figure 1), confirming that both proteins are direct targets of *miR-21*. Furthermore, both PTEN (rs = −0.502; *p* = 0.005) (Figure 3A, 3B) and PDCD4 (rs = −0.426; *p* = 0.019) (Figure 3C, 3D) protein expression significantly and inversely correlated with the expression of *miR-21* in HER2-positive breast cancer patients. However, only PTEN showed a mild association with response to therapy (*p* = 0.033) (Supplementary Table 2). Overall, these findings suggest that the reduced expression of these protein markers alone may not be sufficient to predict response to preoperative trastuzumab and chemotherapy.

**Figure 4:**
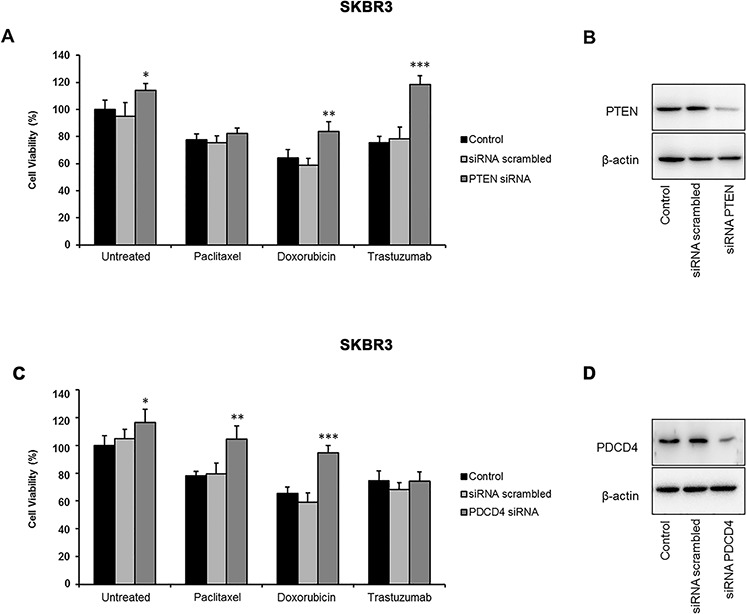
Effects of PTEN and PDCD4 silencing on the viability of HER2-positive breast cancer cells SKBR3 cells were treated with paclitaxel (5 nM), doxorubicin (1 μM), or trastuzumab (10 μg/mL). Cell viability was analyzed using the MTT assay. **A.** PTEN silencing significantly reduced the effect of doxorubicin and trastuzumab. **B.** The efficiency of PTEN silencing was assessed by western blotting. **C.** PDCD4 silencing enhanced the resistance of SKBR3 cells to paclitaxel and doxorubicin. **D.** The efficiency of PDCD4 silencing was assessed by western blotting. Results are presented as percent cell viability relative to controls. Experiments were conducted in triplicate, and error bars represent the S.D. *p < 0.05; **p ≤ 0.01; ***p ≤ 0.001.

To further explore the role of these targets in *miR-21*-mediated drug resistance, we evaluated the effects of PTEN and PDCD4 silencing on the viability of SKBR3 cells treated with paclitaxel, doxorubicin, or trastuzumab (Figure 4). We found that a reduction of PTEN consistently increased the viability of untreated cells (*p* = 0.022) and enhanced the resistance of SKBR3 cells to treatments with doxorubicin (*p* = 0.012) and trastuzumab (*p* = 0.001), but failed to affect the viability of paclitaxel-treated cells (Figure 4A, 4B). Conversely, the downregulation of PDCD4 slightly increased the survival of untreated cells (*p* = 0.036), and significantly reduced the sensitivity of SKBR3 cells to both paclitaxel (*p* = 0.005) and doxorubicin (*p* = 0.001) (Figure 4C, 4D). These results suggest that distinct molecular mechanisms, specifically those affecting the sensitivity to different drugs, may be responsible for the *miR-21*-mediated resistance to trastuzumab and chemotherapy in HER2-positive cancer cells.

### *MiR-21* signaling is involved in an EMT-reinforcing loop that exerts immunomodulatory functions in HER2-positive breast cancer cells upon PI3K pathway activation

**Figure 5:**
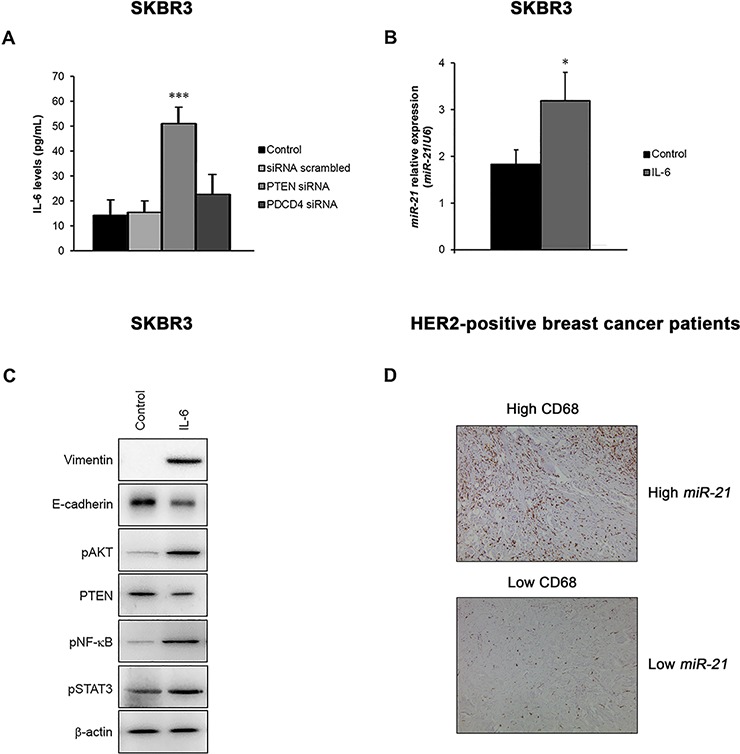
The *miR-21* signaling is involved in an EMT loop that exerts important immunomodulatory functions **A.** The release of IL-6 was consistently increased by the silencing of PTEN in SKBR3 cells. **B.** Treatment of SKBR3 cells with recombinant IL-6 increased the expression of *miR-21*. **C.** IL-6 induced EMT by upregulating specific mesenchymal markers, such as vimentin, and downregulating epithelial markers, such as E-cadherin. Furthermore, IL-6 triggered the activation of the PI3K pathway via the suppression of PTEN and phosphorylation of AKT, which activated an inflammatory signaling cascade by increasing the levels of phosphorylated STAT3 and NF-κB in SKBR3 cells. **D.** Representative images of CD68 protein expression assessed by immunohistochemistry in the validation cohort. Breast cancer samples expressing high levels of *miR-21* showed high CD68 staining (rs = 0.417; *p* = 0.022), supporting the immunomodulatory role of *miR-21*. The data are presented as the mean ± SD of three independent experiments.*p < 0.05; ***p ≤ 0.001.

To further elucidate the functional role of *miR-21* associated with drug resistance, we investigated its involvement in the induction of a local immune response, which may sustain EMT in HER2-overexpressing cancer cells. Our data demonstrated that PTEN silencing, but not PDCD4 silencing, increased the release of IL-6 from SKBR3 cells (*p* = 0.001) (Figure 5A), and that the direct stimulation of cells with IL-6 increased the expression of *miR-21* (*p* = 0.016) (Figure 5B). In addition, IL-6 induced EMT, as indicated by the down-regulation of E-cadherin and the up-regulation of vimentin, activated the PI3K pathway (via the downregulation of PTEN and up-regulation of phosphorylated AKT), and triggered an inflammatory loop by increasing the levels of phosphorylated STAT3 and NF-κB proteins (Figure 5C). Overall, these results suggest that *miR-21* signaling sustains EMT in these cancer cells to cause tumor progression and therapy resistance, and may affect the tumor immune microenvironment by activating IL-6 signaling. In support of these findings, we also found that HER2-positive tumors expressing high levels of *miR-21* showed a significant high content of CD68-positive macrophages (rs = 0.417; *p* = 0.022), supporting the immunomodulatory role of this miRNA (Figure 5D).

## DISCUSSION

The addition of trastuzumab to cytotoxic therapies has significantly improved the response rates and outcomes of HER2-positive breast cancer patients. Despite this success, drug resistance, particularly to trastuzumab, remains an important clinical challenge. Several preclinical models have identified different mechanisms of resistance [1–9]. However, clinical studies have demonstrated the limited value of potential molecular predictors of response or resistance to anti-HER2-based therapies [3, 10]. In this study, we assessed the expression of a subset of specific miRNAs that are involved in the modulation of the tumor-associated immune response and EMT features and evaluated their association with pCR or RD in two independent cohorts of HER2-positive breast cancer patients treated with neoadjuvant trastuzumab and chemotherapy. The expression of a single miRNA, *miR-21*, was specifically higher in patients with HER2-amplified breast cancer with RD compared with patients who achieved pCR, indicating that *miR-21* is an important molecular mediator of drug resistance. Consequently, we demonstrated that the *miR-21* expression levels were further amplified following trastuzumab and chemotherapy, suggesting a possible increase in metastatic potential. Consistent with our findings, chemotherapy-induced DNA damage has been shown to upregulate the expression of *miR-21* by activating NF-κB, which enables breast cancer cells to override DNA damage-induced apoptosis and enhances their invasiveness [28]. Therefore, higher levels of *miR-21* may contribute to both primary and acquired resistance to trastuzumab-chemotherapy treatment in patients with HER2-positive breast cancer. Trastuzumab, either in the early or metastatic setting, is co-administered with cytotoxic chemotherapy. Thus, the identification of reliable markers of trastuzumab resistance should be carefully evaluated, and the presence of high levels of *miR-21* may suggest trastuzumab resistance independently of the administered chemotherapy. Overall, our data indicate that the measurement of the expression of *miR-21* at baseline or in residual cancer after neoadjuvant therapy may be used to understand the likelihood of therapeutic response and to select patients harboring HER2-positive, drug-resistant tumors who may benefit from novel pharmacological agents.

Herein, we confirmed that *miR-21* is involved in EMT and targets PTEN and PDCD4 in HER2-positive breast cancer patients, which corroborates previous findings [13, 29]. Our data support that the *miR-21*-mediated epigenetic silencing of both tumor suppressor genes, particularly PTEN, could be an important mechanism of resistance to trastuzumab-chemotherapy in patients with HER2-positive tumors. As a result, we demonstrated that the downregulation of PTEN and PDCD4 differentially influenced the sensitivity of HER2-positive breast cancer cells to current drugs. Several experimental models have proven that the activation of the PI3K pathway, especially via the loss of PTEN, is responsible to determine resistance to trastuzumab [3–8]. Notably, a lack of PTEN expression only marginally correlated with RD after neoadjuvant trastuzumab-chemotherapy in this study. These findings are supported by recent studies that suggest that a loss of PTEN is associated with poor outcome but has very limited predictive value to measure the response to trastuzumab-based therapy [30, 31]. Potential explanations for these conflicting results are linked to a lack of standardization for PTEN status determination and consequent challenges of reproducibility across different studies, complex mechanisms of drug resistance, and confounding effects from different types of concomitant chemotherapies [3, 5, 10]. These findings, together with the lack of association between PDCD4 and response to trastuzumab and chemotherapy treatment, suggest that either PTEN or PDCD4 alone, likely may not reliably predict the response of HER2-positive breast cancer patients to therapy. Conversely, we demonstrated that the inhibition of *miR-21* synergistically increased the ability of chemotherapeutic agents and trastuzumab to reduce the viability of HER2-positive breast cancer cells. These results indicate that *miR-21* leads to resistance to trastuzumab and chemotherapy via different mechanisms, confirming that *miR-21* could serve as a comprehensive predictive marker of response to such therapy for HER2- positive breast cancer patients.

Despite the important role of *miR-21*-mediated PTEN and PDCD4 deficiency in drug resistance, the response to anti-HER2 and chemotherapeutic agents may be dependent on additional mechanisms, including the boosting of a local inflammatory signaling, which is responsible for the release of specific cytokines and the expansion of tumor cells with an EMT phenotype [13, 18, 28]. Consistently, we found that PTEN silencing increased the production of IL-6, which consequently increased the expression of *miR-21* and induced EMT. Our results are in agreement with previous findings showing that the release of IL-6 by mesenchymal cancer cells and tumor-associated macrophages was able to induce EMT and macrophage polarization toward a pro-tumoral M2 profile [13, 18, 19, 32, 33]. In addition, our *in vitro* assays demonstrated that the silencing of PTEN in HER2-amplified breast cancer cells was indeed able to activate the PI3K pathway and an inflammatory signaling cascade driven by the STAT3/NF-κB pathway. The activation of both PI3K/AKT and STAT3 signaling has been recognized to shape the tumor immune microenvironment and modulate the anti-tumor immune response, which emphasizes the therapeutic potential of targeting both oncogenic and immune checkpoint pathways (*e.g.* PD-1 or CTLA4) in drug-resistant HER2-positive breast tumors [34]. Furthermore, we demonstrated that *miR-21* overexpression correlated with the infiltration of macrophages, suggesting a potential crosstalk between the tumor and immune microenvironment that sustains an EMT phenotype and drug resistance in HER2-positive cancer cells. Our data are reinforced by the association between a low content of macrophages and the response to neoadjuvant therapy, as well as the increased overall survival of HER2-positive breast cancer patients [35]. The infiltration of macrophages is responsible for the recruitment of tumor-infiltrating lymphocytes, which are now emerging as potential prognostic, and possibly predictive, factors in HER2-positive breast cancers [35–38]. Indeed, clinically relevant reciprocal crosstalk between adaptive and innate immune cells, particularly macrophages and T lymphocytes, has been demonstrated in breast cancer, and warrants further study [35, 39].

Collectively, these results suggest that *miR-21* overexpression may be used as a comprehensive predictive biomarker of trastuzumab-chemotherapy resistance and that patients harboring HER2-positive tumors expressing high levels of *miR-21* may benefit from the addition of PI3K inhibitors or immunomodulatory drugs to current anti-HER2 therapies.

## MATERIALS AND METHODS

### Patients characteristic and tumor samples

Formalin-fixed, paraffin-embedded (FFPE) core biopsies were retrospectively collected from 52 patients harboring histologically confirmed HER2-positive breast cancer who underwent neoadjuvant treatment consisting of trastuzumab and chemotherapy at Vall d'Hebron University Hospital and Humanitas Clinical and Research Institute from 2005 to 2011. Paired post-chemotherapy specimens were also available for 12 of these samples. Ethical approval was obtained from the review boards at the Spanish and Italian Institutions. Tissues from 21 HER2-negative breast cancer patients who underwent neoadjuvant anthracycline- or taxane-based chemotherapy were also collected and used as controls. The patient characteristics are presented in Supplementary Table 1. HER2-overexpressing breast cancer patients were divided into a discovery (*n* = 22) and a validation (*n* = 30) cohort. Response to therapy was dichotomized as pCR (defined by the absence of a residual invasive tumor in the breast and axillary lymph nodes) or RD. Among the 52 HER2-positive patients, 28 patients achieved a pCR, whereas the other 24 cases had RD (Supplementary Table 1).

### RNA isolation and quantitative real-time PCR analysis

Total RNA from two 6-μm FFPE sections was extracted using a High Pure miRNA Isolation Kit (Roche, Milan, Italy) based on the company's specifications. The extracted RNA was then eluted in RNase-free water and stored at −80°C until use. Based on a literature review, we selected the most functionally relevant miRNAs associated with immune function and EMT regulation (*let-7b-3p*, *miR-18b-3p*, *miR-20a-3p*, *miR-21–5p*, *miR-34a-5p*, *miR-106b-3p*, *miR-107*, *miR-145–3p*, *miR-155–5p*, *miR-181b-5p*, *miR-200b-3p*, *miR-221–3p*, *miR-373–3p* and *miR-495–3p*) and evaluated their expression in 22 HER2-positive (discovery cohort) and 21 HER2-negative core biopsies by quantitative real-time PCR (qRT-PCR). Briefly, total RNA was reverse transcribed using the miRCURY LNA™ Universal RT microRNA PCR kit (Exiqon, Vedbaek, Denmark). The expression of each miRNA was assayed on the microRNA custom Pick-&-Mix panel (Exiqon) in a LightCycler 480 Real-Time PCR System (Roche). Negative controls without template and the cDNA synthesis control (UniSp6) were included. All data were normalized to the average of assays detected in all samples (average Ct-assay Ct). The two miRNAs expressed in all samples (*miR-16–5p* and *let-7a-5p*) were used to normalize miRNA expression. Upon the selection based on a statistical analysis, the expression of *miR-21–5p* was further analyzed in the validation cohort (*n* = 30). Briefly, 20 ng of total RNA from each sample was reverse transcribed using the TaqMan MicroRNA Reverse Transcription kit (Life Technologies, Monza, Italy). Each amplification reaction was performed in triplicate. The miRNA expression was quantified in relation to the expression of small nuclear U6 RNA. The relative expression of *miR-21* was calculated using the comparative Ct method.

### Immunohistochemistry

The 30 samples of the validation cohort were subjected to immunohistochemistry (IHC). FFPE sections (3 μm) were deparaffinized and subjected to epitope retrieval. Tissue sections were treated with Peroxidase Blocking Reagent (Dako, Milan, Italy) and Background Sniper (Biocare, Milan, Italy) and then incubated with antibodies to PTEN (Cell Signaling, Danvers, USA), PDCD4 (Abcam, Cambridge, UK), and CD68 (Dako). Negative control slides without primary antibody were also included in the analysis. Immunodetection was performed with the Envision Plus Horseradish Peroxidase Kit (Dako) and diaminobenzidine (DAB; Biocare) was used for colorimetric visualization followed by counterstaining with hematoxylin. The PTEN and PDCD4 expression levels were semi-quantitatively scored based on the staining intensity (0 = negative; 1 = weak; 2 = moderate; and 3 = strong) and the percentage of positive cells (0 < 1%; 1 = 1–25%; 2 = 26–50%; 3 = 51–80%; and 4 > 80%). Ten visual fields from different areas of each tumor were used to evaluate the IHC staining. A composite immunoreactive score (IRS) was calculated by multiplying the intensity by the percentage scores, and the staining intensity was graded as follows: 0 = IRS 0–3 (negative), 1 = IRS 4–6 (weakly positive), 2 = IRS 7–9 (positive), and 3 = IRS 10–12 (strongly positive). Patients were stratified by low (0–1) or high (2–3) PTEN or PDCD4 expression for statistical analyses. The CD68 staining was scored based on the infiltration density of CD68 positive cells, which ranged from 0 (absent) to 3 (dense).

### Cell cultures and treatments

SKBR3 cell lines were obtained from the American Type Culture Collection (ATCC, Manassas, USA). The cells were maintained in RPMI containing 10% fetal bovine serum (FBS) and penicillin/streptomycin (Life Technologies, Monza, Italy) at 37°C in an atmosphere of 5% CO_2_. Trastuzumab was dissolved in sterile water, whereas doxorubicin and paclitaxel were dissolved in dimethyl sulfoxide (DMSO). The cells were treated with paclitaxel (25 nM), doxorubicin (1 μM), trastuzumab (10 μg/mL) or control vehicle. Recombinant IL-6 (R&D Systems, Minneapolis, USA) was used at a concentration of 10 ng/mL.

### Cell lines transfection experiments

Small-interfering RNAs (siRNAs) specific to human PTEN and PDCD4 (Dharmacon, Lafayette, USA) or control siRNA that does not target any sequence in the human genome (scrambled) were used in transient transfection experiments. We optimized the transfection conditions for SKBR3 cells as previously described [40]. Gene silencing was performed in three replicates in 96-well plates under predetermined optimal transfection conditions. Anti-miR-21 inhibitor and its corresponding negative control (anti-NC; Ambion, Life Technologies) were then transfected into SKBR3 cells using Lipofectamine 2000 reagent. The final concentrations of miR-21 inhibitor and anti-NC were 30 nmol/L, and the treatments were started 24 h after transfection.

### Western blotting

The protein extracts were isolated using RIPA buffer containing protease inhibitors (Sigma-Aldrich, Milan, Italy) and resolved by SDS-PAGE. The antibody against anti-PDCD4 was obtained from Abcam. Anti-E-cadherin, anti-vimentin, anti-PTEN, anti-phospho-NF-κB (p65), anti-phospho-STAT3, and anti-phospho-AKT antibodies were purchased from Cell Signaling. The membranes were then incubated with the appropriate HRP-conjugated secondary antibodies (Millipore, Billerica, USA), and the protein signal was detected using a chemiluminescent substrate (Millipore). β-actin was used as a loading control (Santa Cruz, Heidelberg, Germany).

### Cell viability assay

Viable cells were identified using the 3-[4, 5-dimethylthiazol-2-yl]-2,5-diphenyltetrazolium bromide assay (MTT; Sigma-Aldrich). Briefly, cells were plated (3 × 10^3^ cells/well) on 96-well plates and incubated overnight to allow cell attachment. The cells were then treated with paclitaxel, doxorubicin, or trastuzumab for 48 hours. Subsequently, the MTT reagent (5 mg/ml in PBS) was added to each well, followed by incubation for 4 h at 37°C. After the incubation, the MTT crystals in each well were solubilized in DMSO, and the absorbance was read at 560 nm with an iMark plate reader (BioRad, Milan, Italy). All treatments were performed in triplicate, and the cell viability was expressed as a percentage of the untreated control (mean ± S.D.).

### Enzyme-linked immunosorbent assay (ELISA)

The levels of human IL-6 in the serum-free conditioned medium of SKBR3 cultured cells were determined by ELISA using the DuoSet ELISA Development Kit (R&D Systems) following the manufacturer's instructions. All experiments were carried out in triplicate and repeated three times.

### Statistical analysis

The differential expression of miRNAs between patient subgroups was evaluated with a nonparametric Mann-Whitney test, and the Wilcoxon-signed rank test was used to compare matched samples. To evaluate the predictive accuracy of the selected miRNAs, (ROC) curves and areas under the curves (AUC) with a 95% confidence interval were calculated. The association between miRNAs and selected targets was explored with Spearman's correlation analysis. Fisher's exact test was used to compare protein expression levels between groups. All tests were two-sided, and the level of significance was set to *p* < 0.05. Statistical analyses were performed using GraphPad Prism version 5.

## SUPPLEMENTARY FIGURE AND TABLES


