# Supplementary material for: MicroRNA-21 links epithelial-to-mesenchymal transition and inflammatory signals to confer resistance to neoadjuvant trastuzumab and chemotherapy in HER2-positive breast cancer patients
Source: Oncotarget. 2015 Oct 7;6(35):37269–80. doi: 10.18632/oncotarget.5495 (PMC4741929; doi:10.18632/oncotarget.5495)
Supplement: Supplementary file 1 [file oncotarget-06-37269-s001.pdf]

SUPPLEMENTARY TABLES AND FIGURE

Supplementary Table 1: Patient characteristics

Clinical and pathological information	HER2-positive		HER2-negative
	Discovery cohort	Validation cohort	
**Samples (*n*)**	22	30	21
**Grade**			
grade 1	–	3.3%	14.3%
grade 2	50.0%	50.0%	76.2%
grade 3	40.9%	43.4%	9.5%
Unknown	9.1%	3.3%	–
**Neoadjuvant chemotherapy**	100%	100%	100%
**Type of neoadjuvant chemotherapy**			
Anthracyclines + Taxanes + Trastuzumab	72.7%	73.3%	–
Taxanes + Trastuzumab	27.3%	26.7%	–
Anthracyclines + Taxanes	–		90.5%
Anthracyclines	–		9.5%
**Response to neoadjuvant chemotherapy**			
pCR	54.5%	53.3%	23.8%
RD	45.5%	46.7%	76.2%

pCR: pathological complete response; RD: residual disease

Supplementary Table 2: PTEN and PDCD4 expression levels in HER2-positive breast cancer patients who achieved pathological complete response and patients with residual disease

	High PTEN	Low PTEN	FET *p*-value	High PDCD4	Low PDCD4	FET *p*-value
**pCR group**	10	6	**0.033**	9	7	0.072
**RD group**	3	11		3	11	

FET: Fisher's exact test
Significant *p*-values are given in bold.

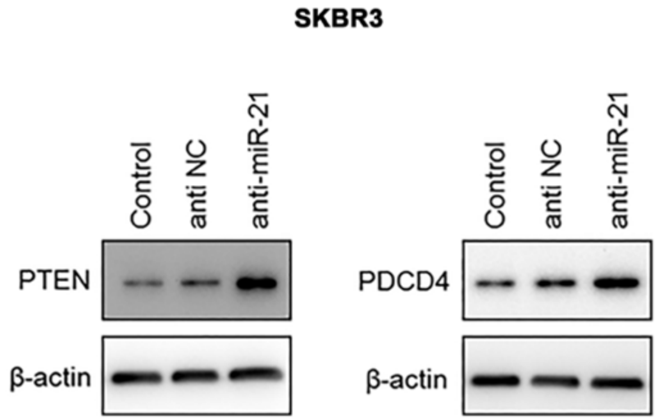

Supplementary Figure 1: PTEN and PDCD4 are direct targets of *miR-21*. The inhibition of *miR-21* was able to increase PTEN and PDCD4 expression in SKBR3 cells.
